# Incorporating distance metrics and temporal trends to refine mixed stock analysis

**DOI:** 10.1038/s41598-022-24279-2

**Published:** 2022-11-29

**Authors:** Gustavo D. Stahelin, Eric A. Hoffman, Pedro F. Quintana-Ascencio, Monica Reusche, Kate L. Mansfield

**Affiliations:** University of Central Florida, 4000 Central Florida Blvd, Orlando, FL USA

**Keywords:** Conservation biology, Ecological genetics, Genetic markers, Population genetics

## Abstract

The distribution of marine organisms is shaped by geographic distance and oceanographic features like currents. Among migratory species, individuals from multiple populations may share feeding habitats seasonally or across life stages. Here, we introduce a modification for many-to-many mixed stock models to include distance between breeding and foraging sites as an ecological covariate and evaluate how the composition of green turtle, *Chelonia mydas*, juvenile mixed stock aggregations changed in response to population growth over time. Our modified many-to-many model is more informative and generally tightens credible intervals over models that do not incorporate distance. Moreover, we identified a decrease in genetic diversity in a Florida nesting site and two juvenile aggregations. Mixed stock aggregations in central Florida have changed from multiple sources to fewer dominant source populations over the past ~ 20 years. We demonstrate that shifts in contributions from source populations to mixed stock aggregations are likely associated with nesting population growth. Furthermore, our results highlight the importance of long-term monitoring and the need for periodical reassessment of reproductive populations and juvenile aggregations. Understanding how mixed stock aggregations change over time and how different life stages are connected is fundamental for the development of successful conservation plans for imperiled species.

## Introduction

Dispersal shapes species' distributions and genetic structure; organisms dispersing into new areas may select suitable habitats based on factors such as availability^1^, temperature^2^, resources, and competition^3,4^. Among mobile organisms, some have defined home ranges and low dispersal, such as the maned sloth (*Bradypus torquatus*)^5^, while others, such as the saltwater crocodile (*Crocodylus porosus*), are distributed more broadly and travel hundreds of kilometers for food and reproduction^6^. Particularly among migratory organisms, individuals originating from multiple populations may share the same habitat and resources (e.g., fishes^7^, and whales^8,9^). These shared habitats can be occupied by individuals from different populations during a specific time of the year, as in gray whales (*Eschrichtius robustus*) that seasonally share foraging habitats in the Pacific Ocean^8^, or during multiple years as in juvenile sea turtle foraging aggregations^10^.

In organisms for which different populations share a habitat, mixed stock analysis (MSA) is a useful technique to understand genetic connectivity between source populations and mixed stock aggregations^11^. There are different approaches for mixed stock calculations, most commonly using maximum likelihood^12,13^ or Bayesian inference^14–16^. In addition, there are models designed to use haploid or single-parent inherited markers (e.g., mitochondrial DNA [mtDNA])^14,17,18^ and nuclear or diploid markers (e.g., microsatellites, allozymes, minisatellites, among others)^12,13,15^. Regardless of the approach, such methods usually compare genetic marker frequencies from mixed stock aggregations to known reproductive populations to estimate contributions to mixed stocks^16^. For mtDNA and similar markers, Bolker et al.^14^ introduced a model that considers the contributions from all available source populations to multiple mixed stocks simultaneously (e.g., many-to-many models). Many-to-many models allow robust and more realistic estimates of source populations than previous models that only accepted one mixed stock aggregation as a possible destination (e.g., many-to-one models^14,16^). Despite the important methodological advancements introduced by Bolker et al.^14^, the many-to-many model introduced in the R package ‘mixstock' was not designed to consider site-specific variables such as the distance between source and destination sites. Nishizawa et al.^19^ added a matrix of distances between source populations and mixed stocks as priors to the model as a means to account for distance in a many-to-many framework; however, it is unclear how their distance matrix is incorporated into the model.

Understanding how demographic parameters and dispersal patterns impact mixed stock composition is fundamental for implementing conservation plans for endangered and threatened species, especially if such variables change over time. Under scenarios where individuals are free and capable of moving in any direction, we would expect large source populations to contribute more individuals to mixed stocks than small populations. However, most organisms disperse between habitats according to biogeographical and resource constraints^1,3,4^. Also, temporal changes in source population sizes and stochastic events altering dispersal patterns can hamper our ability to characterize source populations for mixed stocks^16^. A mixed stock model implemented by Okuyama and Bolker^18^ weights model contribution estimates based on the size of each source population. Mixed stock models often require robust sampling from all sites under evaluation to obtain reliable estimates^17^, leading to combined datasets from previously published studies, regardless of the time period when samples were collected^7,9,10,20–23^. A concern with such an approach is that source populations are not necessarily constant over time^24,25^, just as haplotype frequencies in mixed stock aggregations might fluctuate^26^. Furthermore, the distance traveled by individuals from source populations to foraging aggregations is, in general, an important variable impacting dispersal^27,28^, and is often not considered in mixed stock assessments^7–11,29^ (but see^19–21^). Therefore, there is a need to evaluate the impact of temporal changes in reproductive population demographics on mixed stock aggregations and to develop models that can better account for the distance between breeding and mixed stock aggregations into many-to-many MSAs.

The green turtle (*Chelonia mydas*) is an ideal organism to evaluate how demographic variations and distance between source populations (rookeries) impact mixed stock aggregations. First, green turtle foraging aggregations are typically composed of individuals from multiple populations^10,14,22,23,26,29^. Second, in the past two decades, the number of nests in several green turtle rookeries in the Greater Caribbean and the western North Atlantic have increased at different rates^24,25^. Recently, van der Zee et al.^22^ suggested that changes in contributions observed in a juvenile mixed stock in Bonaire could be associated with a variation in the size of the source nesting populations. Third, green turtles leave nesting beaches as hatchlings and swim away from the coast to offshore habitats where they reside for a number of years^30^. Even though oceanic-stage green turtles are not complete passive drifters and may actively swim and orient^31^, there is substantial evidence suggesting marine turtle juvenile dispersal is also influenced by oceanographic currents, especially during the first few years of their life cycle^32–34^ (but see^35^). Therefore, we can approximate the distance traveled by individuals between rookeries and mixed stock areas by following the main marine currents connecting the different areas. Lastly, the east coast of central Florida, USA, hosts one of the largest nesting aggregations for green turtles in the western North Atlantic^25^ and several mixed stock aggregations^36,37^.

Here, we evaluate the impact of temporal changes in rookery sizes and in green turtle mixed stock aggregations in the Greater Caribbean and western North Atlantic while accounting for distance traveled between rookeries and mixed stock aggregations. To achieve these goals, we (*i*) modify the many-to-many mixed stock model to weight estimates based on the distance between rookeries and mixed stocks, and use the modified model to (*ii*) evaluate how variations in rookery sizes impacted MSA estimates over a two-decade period, and (*iii*) assess how MSA estimates change in response to variation in mixed stock haplotype frequencies and rookery size over the same period.

## Methods

### Study site and data collection

Adding to the available data on haplotype frequencies for rookeries and mixed stocks (Supplementary tables S1–S3), we collected data from nesting female green turtles in the Brevard County portion of the Archie Carr National Wildlife Refuge, in Melbourne Beach, Florida, USA (28.04° N, 80.55° W to 27.87° N, 80.45° W—hereafter referred to as “MB”)^38^. We sampled juveniles at two mixed stock foraging sites: the Indian River Lagoon about two kilometers south of the Sebastian Inlet (27.82° N, 80.43° W—“IRL”), and at Trident Basin at Port Canaveral (28.42° N, 80.59° W—“TRID”), both on the east coast of central Florida, USA^36,37^. All specimens used in this study were collected in accordance with animal care and use protocols approved by the Institutional Animal Care and Usage Committee at the University of Central Florida (IACUC 2020-04, 2020-18, 2020-138, and their predecessors). Skin and blood samples collection were conducted under permits MTP-231, NMFS 19508, and their predecessors.

We defined two sampling periods, “old” and “new”, within the rookery and mixed stock samples: for the rookery, samples collected before 2000 = MB_old_, and samples collected in 2016 to 2018 = MB_new_. At the mixed stock sites, samples from 2003 to 2005 = IRL_old_ and TRID_old_, while samples from 2016 to 2018 = IRL_new_ and TRID_new_. Nesting female samples were assigned to a sampling period based on their first encounter, while juvenile mixed stock samples were assigned to a sampling period if any of the capture dates occurred during the years examined in this study. We recorded the standard straight carapace length (SCL) from the nuchal notch to the tip of the longest pygal scute when possible^39^. We extracted DNA from either skin or blood samples. Skin samples were collected using a 4-mm biopsy punch and stored in 95% ethanol at room temperature. Blood samples were collected from the dorsal cervical sinus into vials with sodium or lithium heparin, centrifuged to separate plasma, and red blood cells were frozen at −20 °C. For most of the blood samples collected from nesting females before the year 2000, a subset of the whole blood was also stored at room temperature in lysis buffer (100 mM Tris–HCl, 100 mM EDTA, 10 mM NaCl, 1% SDS, pH 8.0) using a 1:10 ratio of blood to buffer^40^.

### Laboratory analyses

We extracted genomic DNA using either a Qiagen DNeasy blood and tissue kit following the manufacturer’s protocol or a Serapure Bead method with adaptations^41,42^. We used primers LCM15382^43^ and CM16437^29^ to amplify an 829 bp fragment of the mitochondrial control region (mtDNA). We used 20 µL polymerase chain reactions with final concentrations of 20 mM Tris HCl pH 8.4, 50 mM KCl, 0.25 mM of each dNTP, 1.5 mM MgCl_2_, 0.5 µM of each primer, 1 unit of *Taq* DNA polymerase, approximately 10 ng of genomic DNA, and water. We set up thermal cyclers to the following conditions: 95 °C for 5 min; 40 cycles of 95 °C for 30 s, 57 °C for 30 s, 72 °C for 80 s; and a final extension at 72 °C for 10 min; holding at 10 °C. Samples with haplotype CM-A1.1 were screened at one additional locus using primers CM12751F and CM13064R following PCR and sequencing conditions described in Shamblin et al.^23^. We purified all PCR reactions using Exonuclease I (EN0581) and FastAP (EF0651) following the manufacturer’s protocol. Samples were sequenced in both directions through Sanger sequencing.

### Data analyses

We edited, assembled, and aligned mtDNA sequences to reference haplotypes (829 bp) available from the Archie Carr Center for Sea Turtle Research database (https://accstr.ufl.edu/resources/mtdna-sequences/) using Geneious R8^44^. We created a median-joining haplotype network using PopART v1.7^45^, and calculated pairwise fixation indexes (*F*_ST_), nucleotide (π) and haplotype (*h*) diversities using Arlequin v3.5.2.2^46^. We used *F*_ST_ thresholds proposed by Wright^47^ to assess population differentiation. We compared genetic variation over time for each sampling site via analysis of molecular variance (AMOVA) with 10,000 permutations in Arlequin.

### Mixed stock analysis

**Figure 1:**
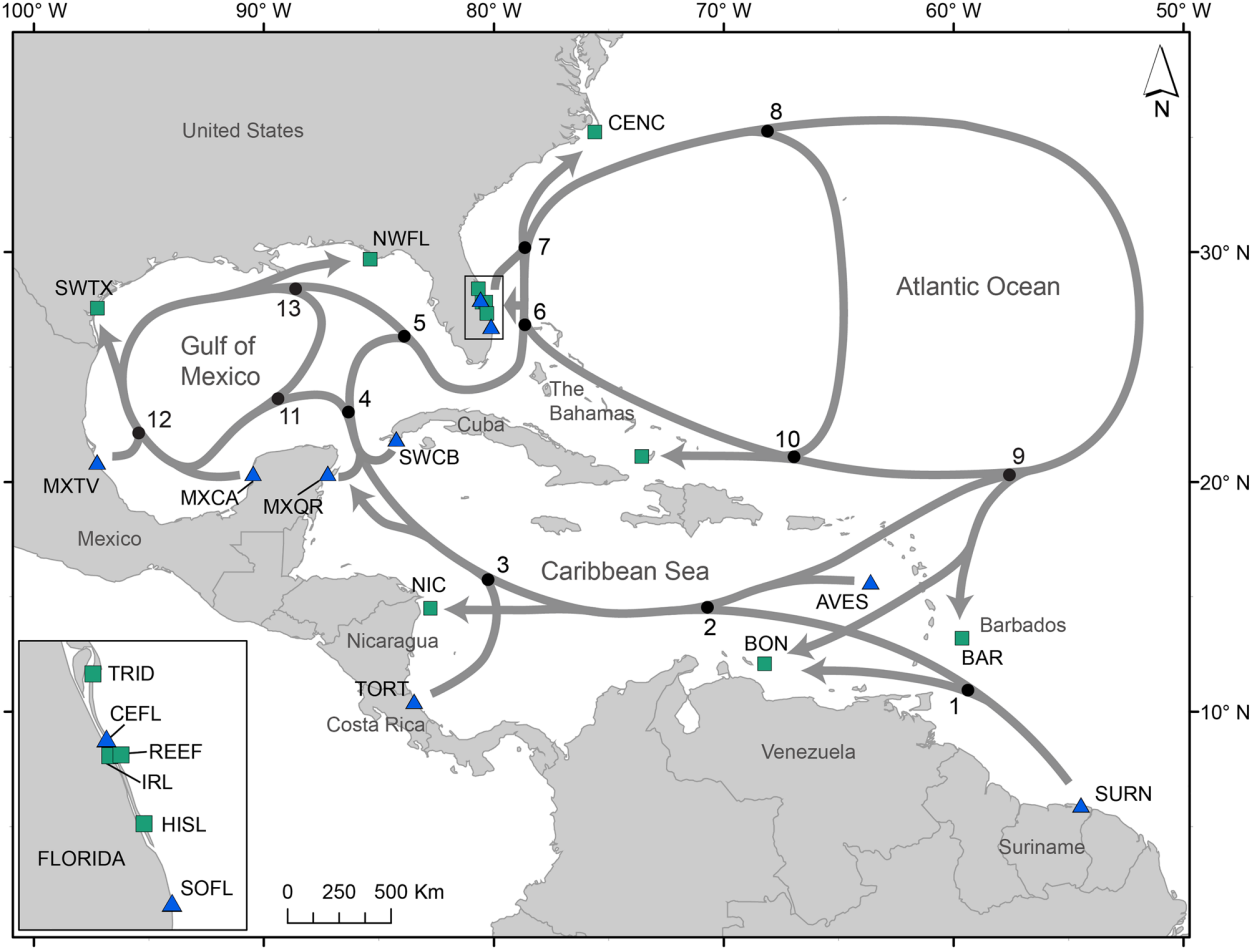
Probable routes used by juvenile green turtles to estimate the relative distances between rookeries and foraging areas; general current direction indicated by gray arrows. Blue triangles are rookeries, and green squares are mixed stock aggregation areas. Samples from MB are part of the CEFL rookery. Rookeries: *AVES* Aves Island, Venezuela, *SURN* Matapica and Galibi, Suriname, *TORT* Tortuguero, Costa Rica, *MXQR* Quintana Roo, Mexico, *MXCA* Campeche and Yucatán, Mexico, *MXTV*  Tamaulipas and Veracruz, Mexico, *SWCB* Guanahacabibes Peninsula, Cuba, *SOFL* South Florida, United States, *CEFL* Central Florida, United States. Mixed stocks: *BAR* multiple areas, Barbados, *BON* Lac Bay, Bonaire, *NIC* Northeast Nicaragua, Nicaragua, *BAH* Southern Bahamas, The Bahamas, *SWTX* Southwest Texas, United States, *NWFL* Northwest Florida, United States, *HISL* Hutchinson Island, United States, *RSBI* Reef at Sebastian Inlet, United States, *IRL* Indian River Lagoon, United States, *TRID* Trident Basin, United States, *CENC* Central North Carolina, United States. Map generated in ArcMap 10.8.1 (https://www.esri.com), and arrows and labels were added in Adobe Illustrator 24.3 (https://www.adobe.com).

We modified the many-to-many mixed stock model, originally implemented in the R package ‘mixstock'^14^. The original model available in the ‘mixstock' package accepts a covariate to weight estimates obtained from haplotype frequency data by the relative size of each rookery^14,18^. Although rookery size is an important factor influencing contributions from rookeries to mixed stocks, the current model does not accept site-specific factors such as distance between rookery and mixed stock location, or main marine currents in between. To date, researchers need to input a matrix of values as priors into the many-to-many model in order to add the effect of distance on estimates^19^. Our assumption is that rookeries might have greater contributions to relatively closer mixed stocks than to distant ones. Similarly, dispersal from rookeries to some mixed stock aggregations can be facilitated by oceanographic conditions. Even though juveniles are capable of orienting and actively swimming in marine currents^31^, there is a greater chance for individuals to disperse to areas closer to where currents initially lead them than to other locations. Following this rationale, we modified the many-to-many model to weight the expected contributions by the scaled inverse distance (*P*) between each source population and mixed stock pair. The model we introduce here is:

$$Estimate\,\sim \,SourceContribution \, * \, SourceSize \, * \, P$$

where *SourceContribution* is the estimated contribution from each rookery based on haplotype frequencies, and *SourceSize* is the estimated size of each source population. The modified model differs from Bolker et al.^14^ only by the scaled inverse distance (*P*) matrix. The code and rationale for the base model with *SourceContribution* and *SourceSize* are described in Bolker et al.^14^ and Okuyama and Bolker^18^. See Supplementary Document S1 for details on our modifications. Here, we populated the matrix with values derived from the estimated inverse distances between each rookery and mixed stock by measuring the length of probable paths between sites using available marine currents as vectors for transport between rookeries and mixed stock aggregations (Fig. 1; Supplementary Tables S4 and S5). Scaled estimates of effective inverse distance (*P*) between each pair of mixed stock (*i*) and rookery (j) were calculated by

1 $${P}_{ij}= \frac{1}{Dij}/ {\sum }_{j=1}^{n}\frac{1}{Dij}$$

where *D* is the estimated distance from the rookery *j* to the mixed stock *i*. Given that a variety of factors may influence the direction and intensity of marine currents^48^, we considered two different scenarios (Scenarios 1 and 2) in which individuals may take different paths to move between sites (Fig. 1—our discussion focuses only on Scenario 1. See Supplementary Table S4 for distance scenario 2). Our goal was to introduce a tool to improve future mixed stock analysis, not necessarily to define dispersal patterns for green turtles.

We used a short fragment of the mtDNA (491 bp) for our MSAs (Supplementary Tables S1–S3), which is contained within the longer (829 bp) fragment. We searched the literature for haplotype frequencies in other mixed stocks and rookeries within the western North Atlantic and Greater Caribbean (Fig. 1, Supplementary Tables S1–S3). We removed from our final dataset haplotypes found in mixed stock aggregations but not yet described in rookeries^14^, and considered only rookeries in the northwest Atlantic and the Greater Caribbean to reduce noise from unlikely contributors^49^. Though mixed stock data published by van der Zee et al.^22^ uses a timeframe slightly different than the one from IRL and TRID, we included their data in our dataset evaluating variations in haplotype frequencies to assess possible variations in other sites as well.

For rookery size we used a three-year average of the number of nests laid (Table 1), based on the best available data we had access to. We estimated rookery size for two time periods: historical (~ late 1990s) and recent (early 2010s). Finally, we included only rookeries in the western North Atlantic and Greater Caribbean for which data on the annual number of nests were available for both time periods considered (Table 1).

**Table 1:** Number of green turtle nests (source size) by rookery used in mixed stock models, relative size (proportion) in relationship to other rookeries, and variation of relative size between the two time periods (historical and recent).

Rookeries	Source size		Proportion		
	Historical (period)	Recent (period)	Historical (%)	Recent (%)	Variation (%)
CEFL	1353 (1997–99)^50^	10,129 (2012–14)^50^	1.36	5.00	↑ 3.64
SOFL	827 (1997–99)^50^	6549 (2012–14)^50^	0.83	3.23	↑ 2.40
MXQR	1039 (1999–2001)^51^	11,907 (2012–14)^51^	1.05	5.88	↑ 4.83
MXCA	636 (1999–2001)^52^	11,281 (2012–14)^52^	0.64	5.57	↑ 4.93
MXTV	528 (1999–2001)^52^	10,713 (2012–14)^52^	0.53	5.29	↑ 4.76
TORT	86,667 (1997–99)^53^	129,060 (2012–14)^53^	87.30	63.72	↓ −23.58
SWCB	159 (1997–99)^54^	242 (2010–12)^54^	0.16	0.12	↓ −0.04
SURN	6562 (1987–89)^24^	19,646 (2008–10)^24^	6.61	9.70	↑ 3.09
AVES	1500 (1990s)^55^	3000 (2010s)^55^	1.51	1.48	↓ −0.03

See Fig. 1 for site abbreviations.

We ground-truthed our model to ensure that the modified model results were consistent with the original model in the ‘mixstock’ package when incorporating a matrix of ones (Supplementary Document S2). We also used a simulated dataset to compare the estimates from our modified model to the approach used by Nishizawa et al.^19^ and determine if results were similar. We compared estimates from the original many-to-many model (MSA_1_) to estimates from our modified model including a distance matrix (MSA_2_) to demonstrate how inclusion of a new covariate can impact MSA estimates. For the models described below (MSA_3_-MSA_6_) we populated the distance matrix with values from distance scenario 1 (Supplementary Table S4). To evaluate how changes in rookery sizes impacted MSA estimates, we combined all available data for each mixed stock into a single dataset (Supplementary Table S2) and created one model for each period: MSA_3_—“historical” source size, and MSA_4_—“recent” source size. Finally, to assess how contributions from rookeries changed over time based on mixed stocks haplotype frequencies and rookery sizes, we also built two models: MSA_5_—“old” sampling period and “historical” source size, and MSA_6_—“new” sampling period and “recent” source size. We considered samples from mixed stock BON^22^ to be from comparable timeframes (2006–07 and 2015–16) to IRL and TRID. Therefore, we added haplotype frequencies from BON to our "old" and "new" sampling periods in MSA_5_ and MSA_6_, and used the same haplotypic data from MSA_3_/MSA_4_ for all other mixed stocks (Supplementary Table S3). Even though the IRL, TRID, and BON are the only mixed stocks with data to answer our last goal, we included all other mixed stocks in models MSA_5_ and MSA_6_ to ensure estimates were more accurate. We used three chains for each model with a random starting point. We adjusted the number of iterations and burn-in period for models (Supplementary Table S6) to ensure chain convergence by checking the Gelman-Rubin shrink factor (< 1.08). To determine evidence of changes between models, we compared estimates by subtracting the posterior distributions for each estimated parameter between two models (e.g., MSA_3_ vs MSA_4_). The resulting distribution was used in the Test of Practical Equivalence implemented in the R package ‘bayestestR’^56^ against a null hypothesis (CI = 0.89, range = −0.05 to 0.05). In short, the Test of Practical Equivalence evaluates what proportion of the credible interval of the resulting distribution (i.e., 89% CI) that falls inside the range defined as the null (i.e., −0.05 to 0.05)^56^. We chose a credible interval of 89% based on small posterior distributions sample size (< 10,000)^56,57^. Finally, we used linear regressions to test if the mean estimates from models MSA_3_-MSA_6_ were correlated to the distance between rookeries and mixed stocks.

## Results

### Biometrics

We sampled a total of 200 turtles among the three locations and two time periods (Table 2). The mean SCL of first capture for nesting females in MB_old_ was 100.9 cm (SD 5.1, range 93.4–114.1) and for MB_new_ was 97.8 cm (4.7, 90.5–108.6). For mixed stock samples, the mean size of first capture at IRL_old_ was 46.8 cm (10.8, 29.5–68.6) and for IRL_new_ 48.1 cm (8.4, 32.4–66.7), while TRID_old_ was 29.5 cm (2.9, 23.4–39.2) and TRID_new_ was 30.9 cm (4.3, 23.7–43).

**Table 2:** Haplotypes identified using the 829 bp mitochondrial DNA fragment.

Haplotype	Adults		Juveniles			
	MB_old_	MB_new_	IRL_old_	IRL_new_	TRID_old_	TRID_new_
CM-A1.1	11	9^a^	7	11	13	24
**CM-A1.1.1**	**1**	**1**	**6**	**8**	**6**	**21**
**CM-A1.1.2**	**10**	**6**	**1**	**3**	**7**	**3**
CM-A1.2	5	1	4		2	1
CM-A1.4					1	1
CM-A2.1				1		
CM-A3.1	9	11	13	20	17	11
CM-A5.1			1	2	1	
CM-A8.1			2	1		
CM-A13.1			3			
CM-A16.1			3			
CM-A18.1				1	1	1
CM-A18.2					2	
CM-A26.1				1	2	1
CM-A27.1				1		
CM-A28.1			1		2	
CM-A48.3	2					
Total	27	21	34	38	41	39

MB_old_ (nesting females before 2000), MB_new_ (nesting females between 2016 and 2018); IRL_old_ and TRID_old_ (foraging sites between 2003 and 2005); IRL_new_ and TRID_new_ (foraging sites between 2016 and 2018). Bold rows indicate sequencing of a diagnostic fragment to distinguish between variants of haplotype CM-A1.1.

*IRL* Indian River Lagoon, *Trident* Trident Submarine Basin.

^a^2 samples failed to amplify the diagnostic sequence for CM-A1.1.

### Population structure

We identified four different haplotypes in MB (Table 2, Supplementary Fig. S1), including two samples with CM-A48.3 in MB_old_. This is the first time CM-A48.3 has been identified at a nesting site. The short-fragment version of this variant (CM-A48) had previously only been found in Cuba^58^. We identified haplotypes CM-A27.1 and CM-A28.1 for the first time in a juvenile foraging site on the east coast of Florida. Haplotypes CM-A3.1 and CM-A1.1 were the most frequent both in adult (41.7% and 41.7%) and in-water samples (45.8% and 25.0% in IRL, and 35% and 46.3% at TRID) for both “old” and “new” sampling periods (Table 2, Supplementary Fig. S1).

Results from AMOVAs to determine if genetic diversity changed over time per site indicate that most variation was observed within populations and not over time for all sites. However, we did find that among population variation was greater at the MB site, indicating greater change-over-time than found at the in-water sites (Supplementary Table S7). Haplotype (*h*) and nucleotide (π) diversities decreased for all sites over time. In MB, *h* decreased from 0.709 (SD = 0.047) before year 2000 to 0.567 (0.056) in 2016–2018, and π decreased from 2.252 × 10^–3^ (1.482 × 10^–3^) to 0.757 × 10^–3^ (0.693 × 10^–3^). For mixed stock aggregations, in the IRL *h* varied from 0.8 (0.049) in 2003–2005 to 0.65 (0.063) in 2016–2018 and π from 3.526 × 10^–3^ (2.112 × 10^–3^) to 2.899 × 10^–3^ (1.794 × 10^–3^), while in TRID *h* went from 0.734 (0.05) to 0.553 (0.068) and π from 2.451 × 10^–3^ (1.566 × 10^–3^) to 1.13 × 10^–3^ (0.885 × 10^–3^). The TRID mixed stock saw the highest reduction in haplotype diversity (from 0.732 to 0.553). Despite variation in haplotype and nucleotide diversities, pairwise *F*_ST_ comparisons did not indicate significant variation within sites over time (Table 3). The only differences in *F*_ST_ were between IRL_old_ and both MB sampling periods, between IRL_new_ and MB_old_, and between TRID_new_ and both IRL sampling periods. We found no evidence of structuring between sampling timeframes for each site. For rookery data, we also grouped samples with previous studies for the models evaluating changes in haplotype frequencies (MB is part of the CEFL rookery—Supplementary Table S1).

**Table 3:** Pairwise distance between sampling sites.

	MB_old_	MB_new_	IRL_old_	IRL_new_	TRID_old_	TRID_new_
MB_old_	–	0.710	0.075	0.084	0.710	0.710
MB_new_	0.046	–	0.495	1.000	1.000	0.710
IRL_old_	**0.064**	**0.056**	–	0.930	0.710	0.091
IRL_new_	**0.063**	0.004	0.013	–	1.000	0.132
TRID_old_	0.022	−0.007	0.027	−0.004	–	0.930
TRID_new_	0.033	0.040	**0.077**	0.047	0.011	–

Cells in bold indicate moderate genetic differentiation (0.05 < *F*_ST_ < 0.15)^47^. Cells below diagonal show pairwise *F*_ST_ values. Values above diagonal show estimated p-values obtained from bootstrapping after correcting for multiple comparisons.

### Distance matrix

**Figure 2:**
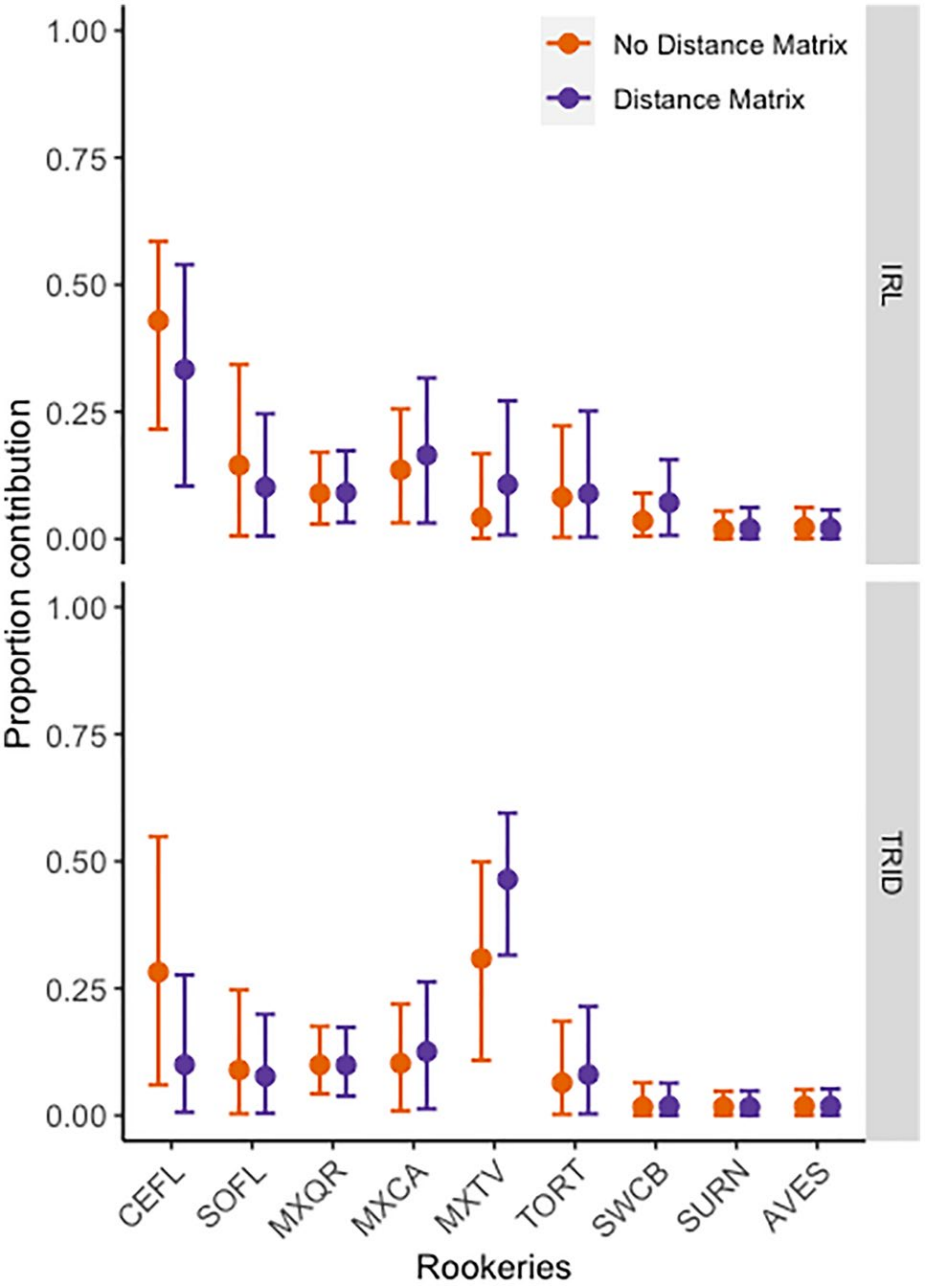
Impact of incorporating a distance matrix into many-to-many mixed stock models. Solid points represent mean estimates and vertical bars 95% credible intervals. No Distance Matrix (MSA_1_) = standard many-to-many model from package ‘mixstock’; Distance Matrix (MSA_2_) = modified many-to-many model with a distance matrix. See Fig. 1 for site abbreviations.

Comparing the estimates obtained using our modified model and the approach used by Nishizawa et al.^19^ we found that our modified model consistently provided estimates with narrower credible intervals than adding distances as priors (Supplementary Fig. S2). Regarding the inclusion of a distance matrix to a many-to-many approach, estimates from rookery SWCB to the TRID mixed stock and from MXQR, SURN, and AVES to both mixed stocks remained essentially the same between MSA_1_ and MSA_2_ (Fig. 2). However, adding the distance matrix in MSA_2_ made the credible intervals wider from CEFL, MXCA, MXTV, TORT, and SWCB to the IRL mixed stock, and from MXCA and TORT to the TRID estimates. In contrast, credible intervals were narrower from SOFL to the IRL mixed stock, and from CEFL, SOFL, and MXTV to the IRL. We found a weak relationship between values populated into the distance matrix and rookeries contribution estimates for models MSA_4_-MSA_6_ (Supplementary Fig. S3).

### Effect of rookery size on mixed stocks

All rookeries showed an increase in the average number of nests per season from historical to recent time periods (Table 1). However, given the different rates of increase, the relative contribution from each rookery (the number of nests divided by the total number of nests in all rookeries for each time period) changed over time. For AVES and SWCB, their relative proportion remained virtually unchanged (~ 1.5% and ~ 0.14% respectively). On the other hand, TORT had the largest increase in absolute numbers (over 42,000 annually), but its proportion decreased from 87.30 to 63.72%. For CEFL, SOFL, MWQR, MXCA, MXTV, and SURN there was an increase in their relative proportion at similar rates (3.09–4.93%).

**Figure 3:**
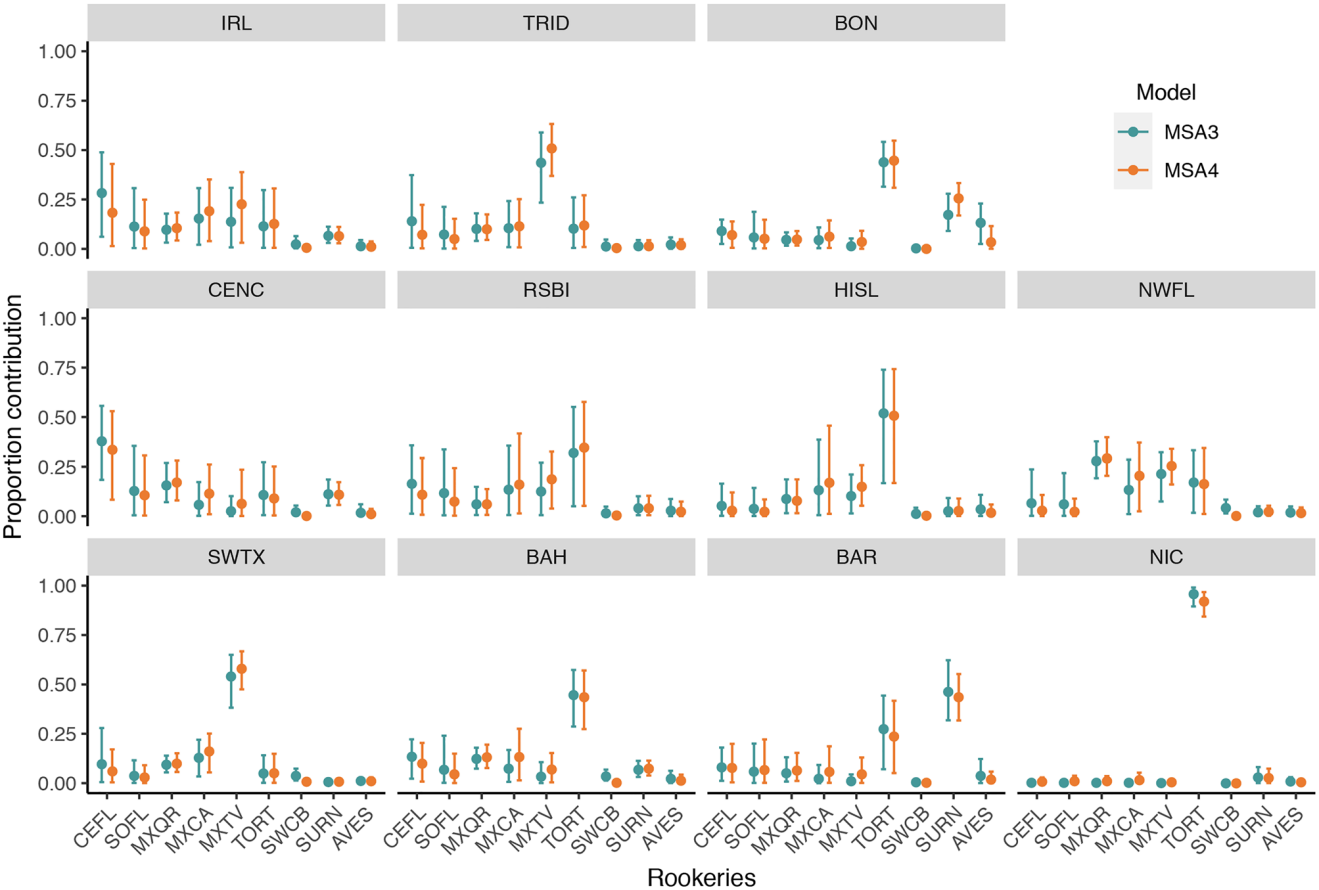
Mixed stock-centric estimates comparing different source sizes. Filled dots represent the mean estimate, and vertical bars 95% credible intervals. *MSA*_*3*_ “historical” source size. *MSA*_*4*_ “recent” source size. Asterisk: rookeries with evidence of a difference in contribution in response to source size variation. See Fig. 1 for site abbreviations.

We found little evidence of changes in contributions to mixed stocks as a response to changes in rookery sizes alone (MSA_3_ vs MSA_4_—Fig. 3). Some mixed stocks analyzed have a single main contributing source population: MXTV is the main contributor to TRID and SWTX, SURN is the main contributor to BAR, and TORT appears as the main contributor to BON, HISL, BAH, and NIC. Contributions to IRL, CENC, RSBI, and NWFL appear to come from multiple sources without a clear single origin. Considering the rookery-centric estimates (Supplementary Fig. S4), individuals from most rookeries disperse similarly among the mixed stocks analyzed (overall mean estimate 8.33%, SD 8.25%). Individuals from TORT disperse mainly to NIC, followed by other mixed stock(s) not present in this analysis (UNK). Main destinations for individuals originating from the SURN rookery were BAR, NIC, and UNK. For both TORT and SURN, there is great uncertainty regarding the main mixed stock destinations. Complete results for models using distance scenario 2 are available in Supplementary Tables S8 and S9.

### Haplotype and source size variation

**Figure 4:**
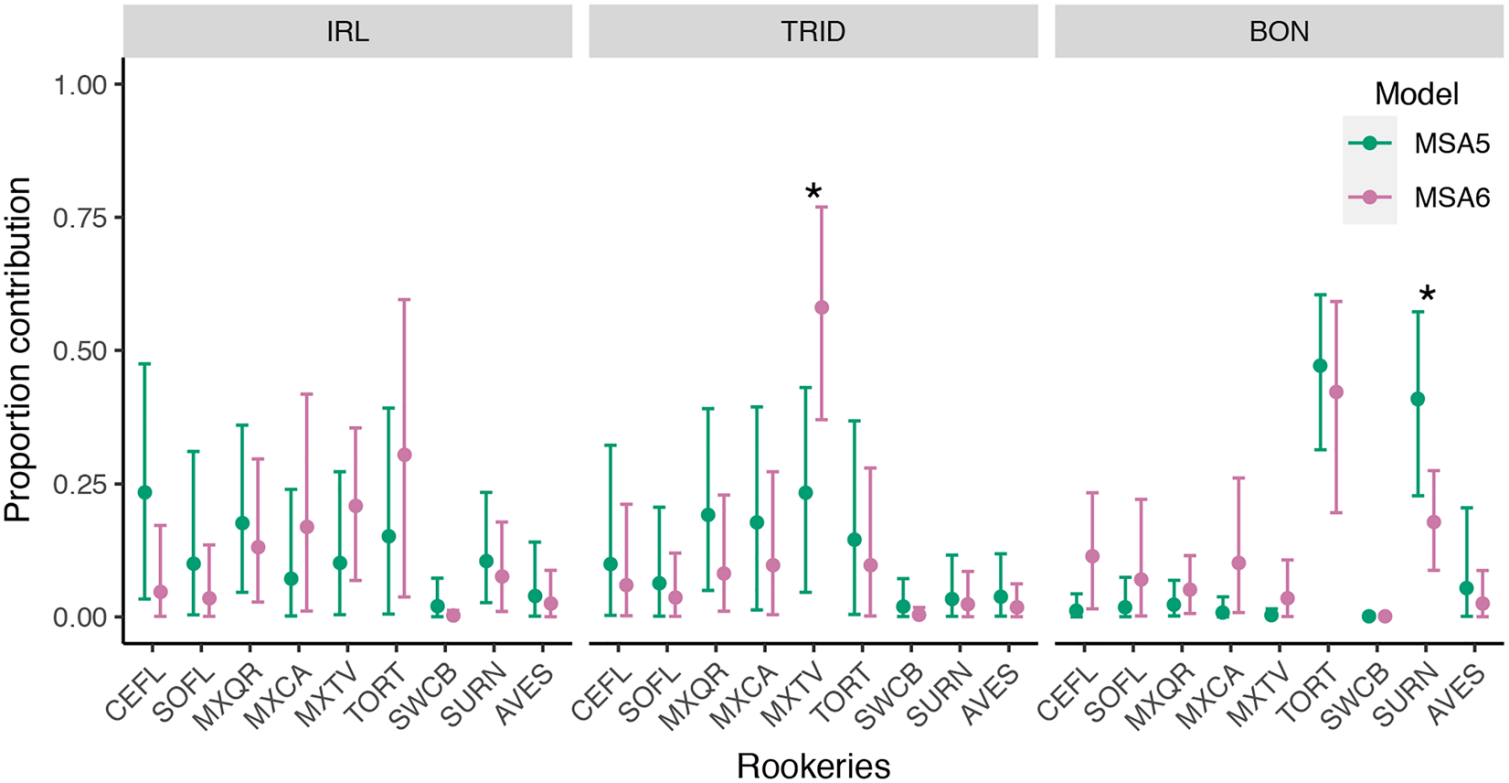
Mixed stock-centric estimates with different sampling events and varying source sizes. Circles represent the mean estimate, and vertical bars 95% credible intervals. *MSA*_*5*_ “old” mixed stock sampling period and “historical” source size. *MSA*_*6*_ “new” mixed stock sampling period and “recent” source size. Asterisk: rookeries with evidence of a difference in contribution in response to source size variation. See Fig. 1 for site abbreviations.

For this objective, we present only the results for mixed stocks with data available for the two sampling periods and the corresponding rookery sizes: IRL, TRID, and BON (MSA_5_ and MSA_6_, Fig. 4—Supplementary Tables S3 and S10 for complete results). The impact of changes in source size on the broader mixed stock estimates was established in the previous section (models MSA_3_ and MSA_4_). For TRID, we found evidence of an increase in the proportion of individuals from MXTV, and for BON there was a decrease in contributions from SURN. Also, for the IRL mixed stock, recent years have narrower credible intervals and lower mean estimates for rookeries CEFL, SOFL, MXQR, SWCB, SURN, and AVES, while wider credible intervals were observed for MXCA and TORT. For TRID, we observed the same pattern of narrower credible intervals for all rookeries except for MXTV. Finally, for BON, recent years appear with tighter credible intervals for SURN and AVES, while for CEFL, SOFL, MXQR, MXCA, MXTV, and TORT we see wider credible intervals (Fig. 4). Source-centric estimates for MSA_5_ and MSA_6_ indicate no changes in destination of individuals from all rookeries over time (Supplementary Fig. S5).

## Discussion

Our study introduces an important advancement for mixed stock analysis: a more informative and ecologically meaningful model incorporating a matrix of site to site-specific weighted inverse distances. We demonstrate how demographic variations in source populations and temporal changes in haplotype frequencies in mixed stocks aggregations can impact MSA estimates. Also, we show how understanding dispersal patterns and connectivity between sites is crucial for management of migratory organisms. Our analyses indicate how the stock composition of juvenile aggregations of green turtles in east central Florida have changed over a 13-year period, simultaneous to a population growth on several source nesting populations. Furthermore, we clearly demonstrate the importance of long-term monitoring and periodic reassessment of both breeding and juvenile aggregations.

For the juvenile IRL and TRID mixed stocks, the mean SCL from individuals sampled in our study was comparable to the mean sizes previously reported for both sites^37,59^. For the rookery MB, the mean size and observed reduction in mean SCL among nesting females are consistent with a trend recently described for this populations^60^. The pairwise comparison between sites (Table 3) corroborates our decision to treat IRL and TRID as separate mixed stocks. Also, F_ST_ indicates a greater genetic differentiation between MB and the IRL mixed stock, suggesting it is not mostly composed of individuals from MB despite geographical proximity, supporting our assumption that the distance between MB and IRL is much greater than what a straight line between these sites suggests (Supplementary Tables S4-S5). Several green sea turtle nesting sites in the western North Atlantic and Greater Caribbean have increased in both estimated abundance and number of nests laid^24^, including the MB nesting aggregation^25^. Female sea turtles are known for reproductive natal philopatric behavior^10^. Given that the genetic marker we used (mtDNA) is both haplotypic and maternally inherited, a reduction in haplotype diversity in reproductive populations would be expected given the reduced effective size associated with mtDNA, especially for historically bottlenecked populations. Regarding in-water aggregations, the observed reductions in *h* and π could be a consequence of changes in the main contributors to each mixed stock (Fig. 4), with a general homogenization of the genetic pool. Similarly, a recent study on a mixed stock in Lac Bay, Bonaire, found a reduction in nucleotide diversity but no clear change in haplotype diversity over 9 years^22^. However, van der Zee et al.^22^ amplified only the short mtDNA fragment, which could reduce their ability to detect variations. Regardless, results from our analysis indicate a predominance of a single contributor in BON in recent years instead of two from the "old" sampling period (Fig. 4), supporting the van der Zee et al.^22^ hypothesis of changes in contributors over time. Even though it is unlikely to be observed on all sites simultaneously, after splitting our dataset for IRL and TRID into two sampling periods, we cannot discard the possibility that these reductions are due to small sample sizes. The reduction in sample size for IRL, TRID, and BON mixed stocks in MSA_5_/MSA_6_ compared to MSA_3_/MSA_4_ could help explain the increased uncertainty around the estimates (Fig. 4). Additionally, we acknowledge that our results are a snapshot in time and encompass less than one generation-time for this species; undetected complex ecological processes might be underway^61^. Future studies with a larger sample size from a single mixed stock and time period could try to address this concern using a resampling approach (e.g., jackknife-based method) to identify how sampling might affect MSA estimates.

We identified variations on the width of credible intervals between our modified model and the original model in the ‘mixstock’ package (MSA_1_ vs MSA_2_). Even though we did not specifically test possible causes for variation in credible intervals after the inclusion of the distance matrix, we suspect it could be related to values in the distance matrix that do not match estimates from haplotype frequencies (e.g., haplotype frequencies indicate small contribution from one rookery while the value in the distance matrix suggest higher contribution from the same source). Mixed stocks and/or rookeries with small sample sizes could be more impacted by such variations.

We found no clear evidence of changes in contributors to mixed stocks when considering variation in rookery size alone (MSA_3_ and MSA_4_; Fig. 3). Previous studies report little or no difference in estimates when comparing models with rookery size versus models with an uninformative covariate (i.e., equal value to all rookeries) while using a many-to-one framework^21,23^. This is not an unexpected result as MSA estimates are mostly derived from genetic markers^16^, and the weight provided by covariates might not be enough to change estimates. However, we found evidence of variations in contributions when the haplotypic variation was considered along with rookery size variation (MSA_5_ and MSA_6_; Fig. 4). Though, we did not test a model with varying haplotype frequencies and constant rookery size, as rookery sizes did change over time this would be an unrealistic scenario and we could not tease these changes apart. Therefore, we cannot determine if the observed fluctuation in haplotype frequencies (and rookery contribution estimates) was caused by changes in the influx of individuals from source populations to mixed stocks or by variation in source population sizes because both possibilities are intrinsically dependent on one another.

The main contributors to mixed stocks from models MSA_3_ and MSA_4_ were partially different from previous analyses in the Atlantic Ocean and Greater Caribbean^14,22,62–69^, which could be explained by substantial differences between our dataset and the different datasets and models used by previous studies. However, results from MSA_5_ and MSA_6_ corroborate findings from studies that identified fluctuations in contributions over time in response to changes in haplotype frequencies in mixed stocks^22,26^. An assumption of mixed stock models is that all source populations are represented and adequately sampled^16^—an assumption that will rarely be met. Engstrom et al.^49^ suggest not including unlikely contributors to mixed stock models to reduce noise, a decision we also made. However, researchers may have different thresholds to define an unlikely contributor, therefore, this decision becomes arbitrary. Comparing estimates among studies is difficult as new areas are added and more samples are sequenced. Furthermore, our modified model uses effective distance to weight estimates; this adds an extra layer of differentiation among studies, making direct study comparisons even harder. Regardless of agreement (or lack of agreement) between our results and previous studies, we believe that future assessments can improve biological meaning if mixed stocks and rookeries are periodically reassessed for haplotype frequencies.

An increase in juvenile abundance following reproductive population growth and increased number of nests laid is a reasonable expectation. This expectation depends on the fitness of reproductive individuals, the hatching success of the nests laid, and survival and recruitment rates for juveniles. However, Bjorndal et al.^61^ found no correlation between increased number of nests at Tortuguero, Costa Rica, the putative main stock of origin for the mixed stock, and variations in the abundance of green turtles at Union Creek, The Bahamas. One hypothesis was that Union Creek was near carrying capacity for green turtles, and abundance would remain stable over time^61^. Our models corroborate their findings, showing TORT as the main contributor (Fig. 3) despite the reduction in TORT’s size in relationship to the other source populations in the region (Table 1). The stability of contributions to BAH could be an indication that the carrying capacity hypothesis is still a valid option for Union Creek. Similarly, Long et al.^59^ attributed a decrease in green turtle abundance in the IRL mixed stock between 2001 and 2018 to a general decrease in habitat quality, despite the increased abundance in rookeries. It is possible that juvenile abundance increased in other mixed stock aggregations and that the observations in the IRL and BAH mixed stocks^59,61^ are isolated cases. However, a study with green turtles from MB identified a decrease in nesting females’ mean size and size at maturity over the past decades, especially after the late 1990s^60^. One of the explanations for a decrease in nesting female body size is reduced juvenile mass growth rate^70^, which, ultimately, could lead to overall reduced reproductive fitness in rookeries. At least for leatherback sea turtles (*Dermochelys coriacea*), reproductive fitness can be impacted by maternal health parameters^71^. Interestingly, these data are supported by our genetic analyses that found little change between in-water sites over time, but greater change among time periods for the nesting beach site.

Mixed stock analysis using either the number of nests or the number of nesting females as a proxy for source size should correct estimates by emergence success (total number of hatchlings emerged divided by the total number of hatched eggs in a clutch). Emergence success can vary among seasons, rookeries, species, and can be affected by maternal health and environmental factors^71,72^. To ensure future mixed stock analyses benefit from more informative rookery sizes, we urge researchers to report the number of nests, hatching success, and emerging success, as well as other basic reproductive parameters from nesting populations. We second the call by Shamblin et al.^73^ for broad use of longer fragments of mtDNA in reassessments of rookeries that have been only evaluated using the short fragment, and especially, that new studies refrain from sequencing the short fragments only. The development of new diagnostic markers using whole mitogenomic sequences^29^, or a combination of mtDNA with other markers (e.g., nuclear microsattelites), to increase discrimination between rookeries is essential for our understanding of sea turtle evolution and dispersal patterns. Future population and species assessments will benefit from better and more refined genetic information.

Understanding dispersal and connectivity among habitats and across life stages is fundamental for species’ conservation. The main feature introduced by our modified model is the capacity for researchers to more easily consider variables that are specific to each pair of source populations and mixed stocks in a many-to-many framework. Prior to our modified model, studies incorporating particle dispersal probabilities or distance between sites often weighted MSA estimates using a many-to-one model framework because the probabilities from a source will differ to each mixed stock, and estimates from multiple mixed stock models need to be combined for a regional overview^20,21,23,33^. Many-to-many models provide estimates with narrower credible intervals than many-to-one models when analyzing the same dataset^14,63,74^. Our modified model usually provided narrower credible intervals than the approach introduced by Nishizawa et al.^19^ on a many-to-many framework. Site-specific probability matrices that incorporate complex variables such as particle dispersal model estimates will enable researchers to consider multiple cohorts, variation within and among seasons, and multiple variables that can impact oceanographic currents^34^.

Our modified many-to-many mixed stock model can incorporate new variables to make models more informative. More importantly, by incorporating distance between rookeries and mixed stocks, or particle dispersal probabilities, models we can better account for unlikely source populations, allowing more realistic estimates of rookery contributions to mixed stocks for robust ocean basin analyses. The short-fragment mtDNA markers used for MSA lack the resolution needed to differentiate between several rookeries^29^. As mixed stock model estimates are mainly derived from haplotype frequencies^14,16^, under scenarios where the genetic marker used is unable to differentiate populations, covariates can help improve model accuracy. The source code and example script for incorporating the site-specific matrix is available in Supplementary Document S1, and we encourage others to use this approach to incorporate distances, transport probabilities, or any other metric that scales the contributions from each rookery to each mixed stock. Contribution estimates from such models will be more ecologically meaningful and more accurate. Further, we highlight the importance of long-term monitoring and periodic reassessment of mixed stock aggregations regarding stocks of origin, abundance, health status, and other population parameters. We also emphasize the importance of periodical reassessment of haplotype frequencies at rookeries, as well as basic demographic and reproductive parameters. For migratory endangered species such as sea turtles, broad analyses considering multiple rookeries within or among ocean basins with more informative estimates are critical for understanding dispersal, connectivity, and evolution. Understanding how the composition of mixed stock aggregations shift over time is fundamental for the development of successful conservation plans for endangered and threatened species.

## Supplementary Information

Supplementary Information.

Supplementary Figures.

Supplementary Tables.

## Data Availability

Data used in mixed stock analyses and code for the modified model with detailed instructions are available as supplementary data.
