# Supplementary material for: Incorporating distance metrics and temporal trends to refine mixed stock analysis
Source: Sci Rep. 2022 Nov 29;12:20569. doi: 10.1038/s41598-022-24279-2 (PMC9709048; doi:10.1038/s41598-022-24279-2)
Supplement: Supplementary file 1 — Supplementary Information. [file 41598_2022_24279_MOESM1_ESM.zip › Supplementary Documents S1-S2/Supplementary Document S2.pdf]

Supplementary Document S2

Article: "Incorporating distance metrics and temporal trends to refine mixed stock analysis"

Authors: Gustavo D. Stahelin, Eric A. Hoffman, Pedro F. Quintana-Ascencio, Monica Reusche, and Katherine L. Mansfield

We conducted the following test to ensure our new model provide contribution estimates comparable to the original many-to-many model from the 'mixstock' package in R.

We generated a template dataset of haplotype frequencies for 3 source populations, 4 mixed stocks, and 10 haplotypes/markers. The number of samples from each source population and from each mixed stock was randomly defined between 25 and 100 samples, and 'source size' was randomly defined between 200 and 500. We used the template dataset generated above to run both a standard many-to-many mixed stock model from package 'mixstock' and our new model with a distance matrix. We would only expect results to be comparable between these two models if all numbers contained in the distance matrix are the same (i.e., distance between all mixed stocks to all source populations is the same). Therefore, we randomly selected a number to feed into the distance matrix between 1 and 100 to account for any variations that could occur with the value used. We confirmed chain convergence using the Gelman-Rubin shrink factor (1) before comparing the results. We repeated this process 100 times to ensure consistency and fitted a linear regression model to assess how estimates of contribution from both models compare. If results are comparable between models, we would expect to see a close-to-perfect model fit ($R^2 \sim 1$). As the mixed stock models use Bayesian inference, small variations around the fit line would are expected.

A compilation of results from the models tested is shown in Fig. 1. The linear regression indicate that our new model provides contribution estimates that are comparable to the original many-to-many mixed stock model, with an $R^2 = 0.997$ for mixed stock-centric estimates and $R^2 = 0.991$ for rookery-centric estimates (Fig. 1).

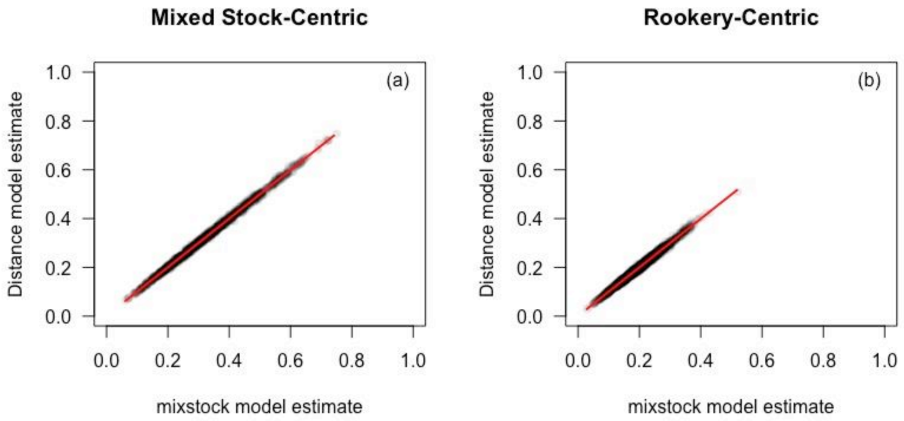

Figure 1: Scatterplots of (a) mixed stock-centric and (b) rookery-centric contribution estimates from the standard many-to-many model from the "mixstock" package and new model introduced by this study with a distance matrix. Red lines indicate the fitted linear regression line.

References

1. Pella J, Masuda M. Bayesian methods for analysis of stock mixtures from genetic characters. Fish Bull. 2001;99(1):151–67.
