# Supplementary material for: Incorporating distance metrics and temporal trends to refine mixed stock analysis
Source: Sci Rep. 2022 Nov 29;12:20569. doi: 10.1038/s41598-022-24279-2 (PMC9709048; doi:10.1038/s41598-022-24279-2)
Supplement: Supplementary file 1 — Supplementary Information. [file 41598_2022_24279_MOESM1_ESM.zip › Supplementary Documents S1-S2/Supplementary Document S1/Supplementary Document S1.pdf]

Supplementary Document S1

Article: "Incorporating distance metrics and temporal trends to refine mixed stock analysis"

Authors: Gustavo D. Stahelin, Eric A. Hoffman, Pedro F. Quintana-Ascencio, Monica Reusche, and Katherine L. Mansfield

The modified model introduced by this paper uses the core functionality and main functions of the 'mixstock' package[1]. Our modification consists of adding a matrix of distances to weight estimates from the model implemented in the 'mixstock' package by distance (or any other matrix of values specific to each pair of mixed stock and rookery), along with a new R function so users can input the new covariate values. The model we introduced is:

*Estimate = SourceContribution[r,m] * SourceSize[r] * **Distance[r,m]***

where *r* is the source population, *m* is the mixed stock, *SourceContribution* is the estimated contribution from each rookery based on haplotype frequencies, *SourceSize* is the estimated size of each source population, and *Distance* is the matrix of values specific to each pair of source populations and mixed stocks. The modified model differs from Bolker et al.[1] only by the *Distance* covariate. The code and rationale for the base model with *SourceContribution* and *SourceSize* are described in Okuyama and Bolker[2] and Bolker et al.[1].

Using this model require users to:

1. Install 'mixstock' package in R (see https://github.com/bbolker/mixstock).
2. Copy the file '*manymany_dist.txt*' to the *~/mixstock/bugs/* directory on your computer*.
3. Load function *mm.wbugs.distance()* on R console (available on file '*mm.wbugs.distance.R' – see below*).

We provide below an example on how to use the modified model in '*Example code - distance model.R'*.

The model in '*manymany_dist.txt*' and function in '*mm.wbugs.distance.R'* are modifications of the original model available in the '*manymany.txt*' and function *mm.wbugs()* from package 'mixstock'[1]. We provide extra files so users can use our modified model while maintaining the core model and functions of the package unaltered.

Additionally, we introduce a new function 'coda_output()' to extract model estimates from MSA models using 'returntype=coda'. See script in file "Example code – distance model.R"

References:

1. Bolker, B. M., Okuyama, T., Bjorndal, K. A. & Bolten, A. B. Incorporating multiple mixed stocks in mixed stock analysis: 'Many-to-many' analyses. *Mol. Ecol.* **16**, 685–695 (2007).
2. Okuyama, T. & Bolker, B. M. Combining genetic and ecological data to estimate sea turtle origins. *Ecol. Appl.* **15**, 315–325 (2005).

---

* To identify the directory where R packages are installed, type on R console:

> *.libPaths()*

Follow the path indicated by the command above, locate the directory *~/mixstock/bugs/* in your computer, and copy the file '*manymany_dist.txt*' into that directory.
Some computers may have more than one directory indicated by *.libPaths()*, for different versions of R. If that is your case, search for the *~/mixstock/bugs/* directory in all indicated paths and copy the file to the appropriate directory.
