# Supplementary material for: Incorporating distance metrics and temporal trends to refine mixed stock analysis
Source: Sci Rep. 2022 Nov 29;12:20569. doi: 10.1038/s41598-022-24279-2 (PMC9709048; doi:10.1038/s41598-022-24279-2)
Supplement: Supplementary file 2 — Supplementary Figures. [file 41598_2022_24279_MOESM2_ESM.pdf]

Supplementary Figures S1 – S5

Article: “Incorporating distance metrics and temporal trends to refine mixed stock analysis”

Authors: Gustavo D. Stahelin, Eric A. Hoffman, Pedro F. Quintana-Ascencio, Monica Reusche, and Katherine L. Mansfield

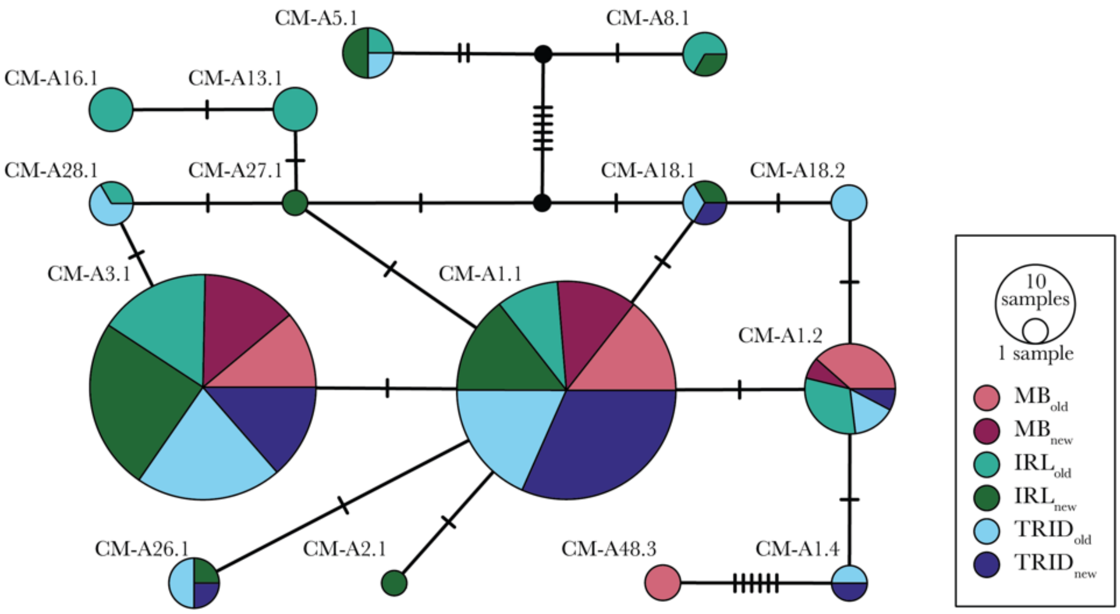

Figure S1: Inferred relationships between haplotypes from nesting and foraging habitats in the East coast of Florida using the median-joining method. Size of each circle corresponds to the frequency of occurrence. MB$_{old}$ (nesting females before 2000), MB$_{new}$ (nesting females between 2016-2018); IRL$_{old}$ and TRID$_{old}$ (foraging sites between 2003-2005); IRL$_{new}$ and TRID$_{new}$ (foraging sites between 2016-2018). Perpendicular bars indicate a 1 bp mutation.

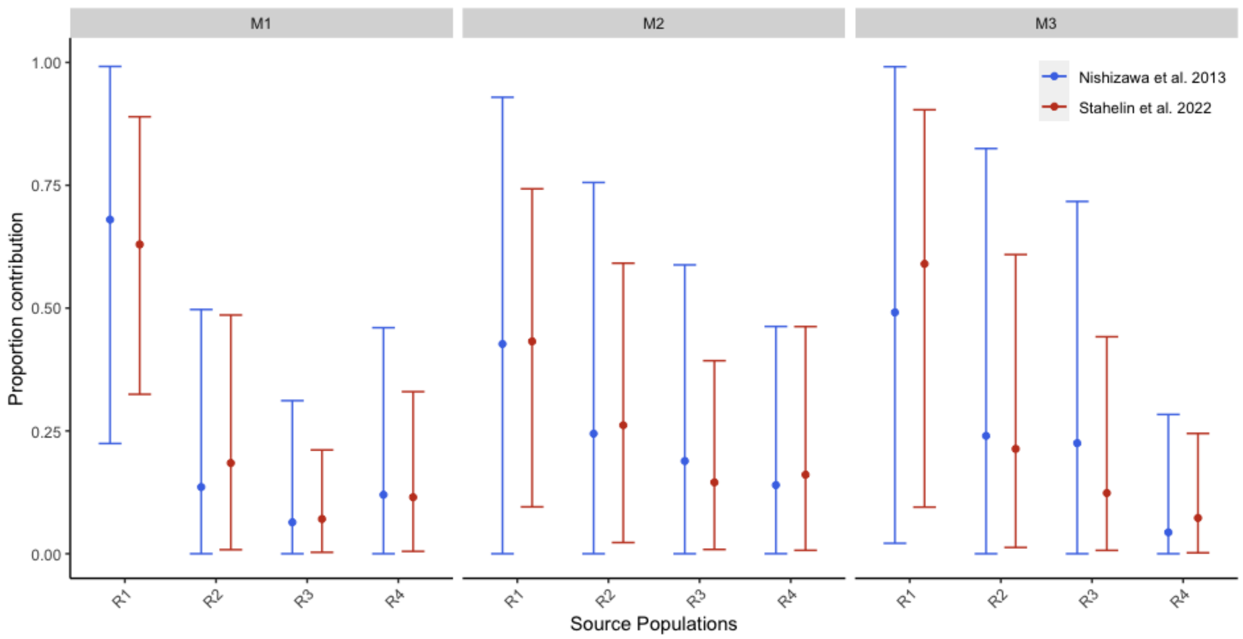

Figure S2: Mixed stock-centric estimates comparing many-to-many approach used by Nishizawa et al. (2013) and our modified many-to-many model. The modified model introduced in this paper consistently provided estimates with narrower credible intervals. Models used the same template data and distance matrix values. Filled dots represent the mean estimate, and vertical bars 95% credible intervals.

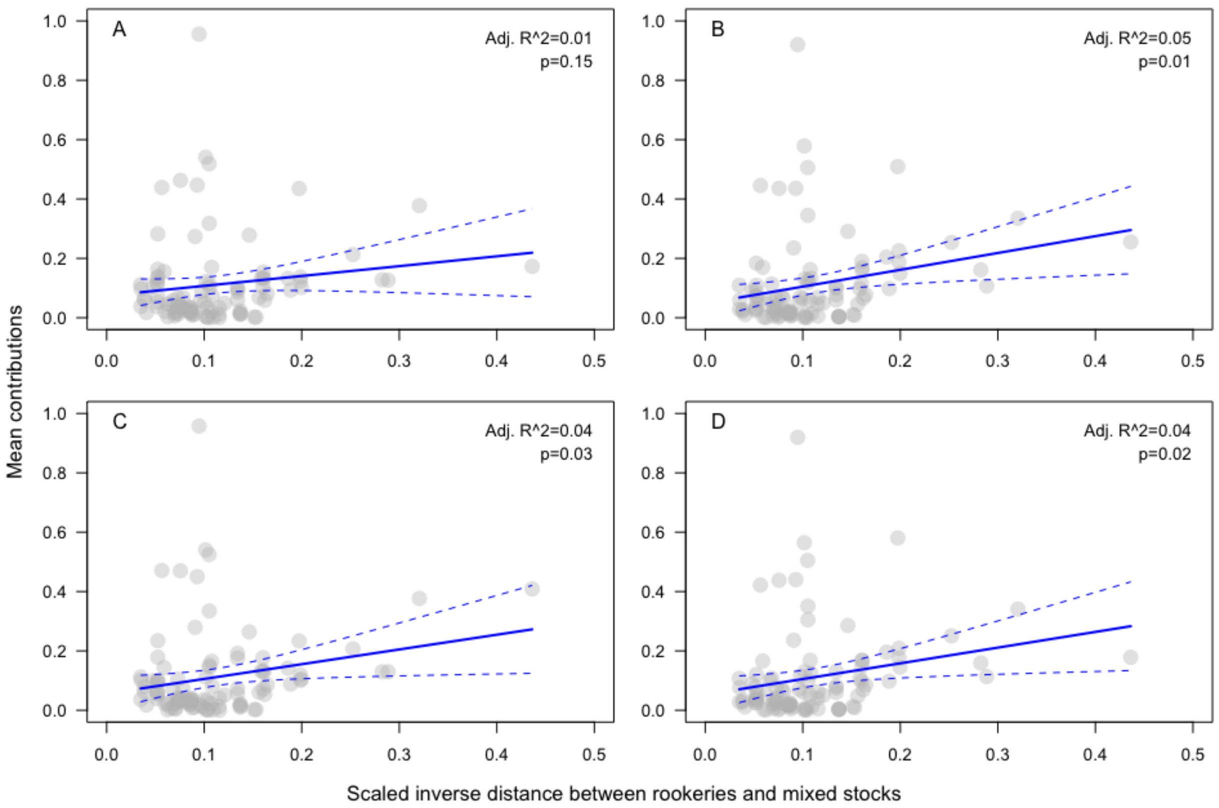

Figure S3: Scatterplot of mean estimates from models MSA_3 (A), MSA_4 (B), MSA_5 (C), and MSA_6 (D) and the scaled inverse distance between rookeries and mixed stocks. Larger scaled inverse values represent smaller distances between rookeries and mixed stocks. Solid line represents predicted values from linear regression models, and dotted lines represent 95% confidence intervals.

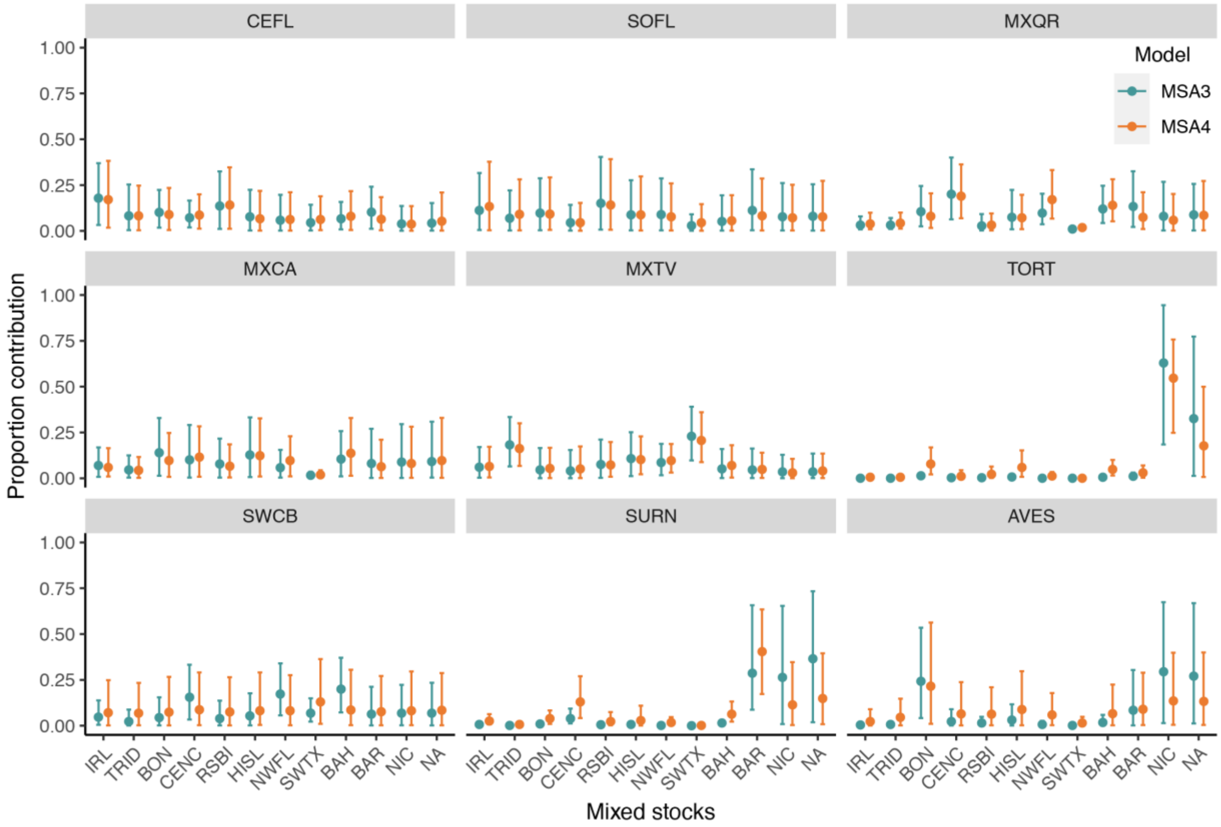

Figure S4: Rookery-centric estimates comparing different source sizes. Filled dots represent the mean estimate, and vertical bars 95% credible intervals. MSA_3 = “historical” source size. MSA_4 = “recent” source size. See Fig. 1 for site abbreviations.

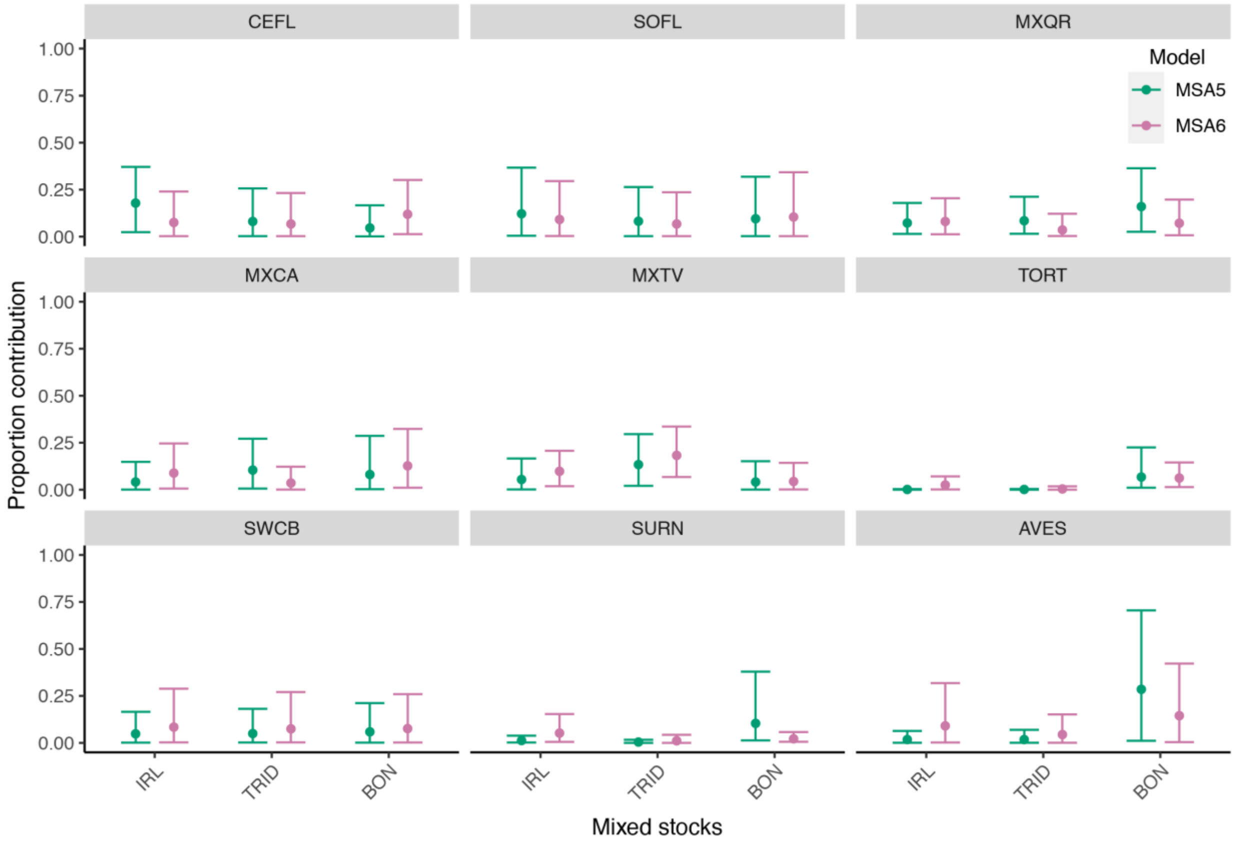

Figure S5: Rookery-centric estimates comparing different mixed stock haplotype frequencies and source sizes. Filled dots represent the mean estimate, and vertical bars 95% credible intervals. MSA_5 = “old” sampling period and “historical” source size. MSA_6 = “new” sampling period and “recent” source size. See Fig. 1 for site abbreviations.
